# 3D Analysis of the TCR/pMHCII Complex Formation in Monkeys Vaccinated with the First Peptide Inducing Sterilizing Immunity against Human Malaria

**DOI:** 10.1371/journal.pone.0009771

**Published:** 2010-03-19

**Authors:** Manuel A. Patarroyo, Adriana Bermúdez, Carolina López, Gloria Yepes, Manuel E. Patarroyo

**Affiliations:** 1 Fundación Instituto de Inmunología de Colombia, Bogotá, Cundinamarca, Colombia; 2 Universidad del Rosario, Bogotá, Cundinamarca, Colombia; 3 Universidad Nacional de Colombia, Bogotá, Cundinamarca, Colombia; University of California Los Angeles, United States of America

## Abstract

T-cell receptor gene rearrangements were studied in *Aotus* monkeys developing high antibody titers and **sterilizing immunity** against the *Plasmodium falciparum* malaria parasite upon vaccination with the modified synthetic peptide **24112**, which was identified in the Merozoite Surface Protein 2 (MSP-2) and is known to bind to HLA-DRβ1*0403 molecules with high capacity. Spectratyping analysis showed a preferential usage of Vβ12 and Vβ6 TCR gene families in 67% of HLA-DRβ1*0403-like genotyped monkeys. Docking of peptide **24112** into the HLA-DRβ1*0401–HA peptide–HA1.7TCR complex containing the VDJ rearrangements identified in fully protected monkeys showed a different structural signature compared to nonprotected monkeys. These striking results show the exquisite specificity of the TCR/pMHCII complex formation needed for inducing **sterilizing immunity** and provide important hints for a logical and rational methodology to develop multiepitopic, minimal subunit-based synthetic vaccines against infectious diseases, among them malaria.

## Introduction

The appropriate fit of antigenic and immunogenic peptides inside the groove or peptide binding region (PBR) of class II major histocompatibility complex molecules (MHCII) is a crucial event for the formation of an appropriate T-cell receptor–(TCR)-peptide–MHCII complex (TCR/pMHCII) and the subsequent activation of an antibody-mediated immune response [1]. Fine antigen recognition and binding specificity is conferred by the interaction of hypervariable amino acid sequences of α and β TCR chains, named **C**omplementarity **D**etermining **R**egions 1, 2 and 3 (CDRs), with structural features of the pMHCII complex, being most diversity concentrated in the β-chain CDR3 [2], [3], [4], [5].

Malaria disease, in particular the one caused by the ***Plasmodium falciparum*** parasite, remains a serious public health problem worldwide, causing more than 500 million cases and killing 3 million of them per year [6]. To develop a fully effective antimalarial vaccine, so desperately needed, it is therefore essential to understand, at the deepest level, the formation of the TCR/pMHCII complex capable of conferring **sterilizing immunity** against this deadly disease.

To develop a logical and rational methodology for designing minimal subunit-based, multiepitopic, multistage, chemically synthesized vaccines, capable of inducing **sterilizing immunity** against this threatening scourge and some others, we have identified functionally-relevant, short (15–20-mer-long synthetic peptides or minimal subunits), conserved High Activity Binding Peptides (HABPs) derived from ***P. falciparum*** proteins involved in invasion to host cells as promising malaria vaccine targets [7].

However, conserved HABPs were found to be neither antigenic nor immunogenic or protection inducers when tested in *Aotus* monkeys, a non-human primate model highly susceptible to human malarias [8], [9] and whose immune system molecules share a high degree of similarity with their human counterparts, specially with those involved in antigen presentation such as α/β TCRs [10], [11] and MHCII–HLA-DRβ1*-like molecules (88% to 100% similarity is reported for the Peptide binding region or PBR of these molecules) [12].

To solve this absence of antigenicity and immunogenicity, hundreds of trials were carried out with native and modified HABPs in large numbers of *Aotus* monkeys, finding that conserved HABPs could be rendered **immunogenic and sterilizing immunity** inducers by replacing their critical host cell binding residues by others having similar mass but opposite polarity. Specific replacement rules were defined [13] such that F must replace R and viceversa (F↔R); W↔Y; L↔H; I↔N; P↔D; M↔K; A↔S; C↔T or V; Q↔E; and G has special physicochemical properties [13], [14].

Bearing in mind these principles, we studied the Merozoite Surface Protein 2 (MSP-2), a 48–69 kDa glycosylphosphatidylinositol (GPI)-anchored merozoite surface molecule considered as promising antimalarial vaccine candidate due to its surface localization and immunological properties [15]. The screening of 20-mer-long peptides spanning the entire sequence of MSP-2 and overlapping with their neighbors by 5 residues led to the identification of the conserved N-terminal HABP 4044 (^21^K**NE**SK**YS**NTF**IN**NA**Y**NMS**I**R^40^), binding with high affinity to red blood cells (RBCs) [16] but same as reported for other conserved HABPs, 4044 was neither immunogenic nor induced protection against experimental challenge with the highly virulent *Aotus*-adapted *P. falciparum* FVO strain when its polymerized form was used as immunogen. Therefore, HABP 4044 peptide analogues modified in critical RBC binding residues identified by glycine analogue screening (highlighted in bold types throughout the manuscript), were synthesized according to the rules mentioned above [17]. Immunization and challenge trials in monkeys lead to the identification of peptide **24112** (^24^SK**YS**NTF**NI**NA**Y**NMV **I**RRSM ^43^; modified residues are shown underlined) as the most promising subunit vaccine component since it induced high antibody titers that recognized a *P. falciparum* merozoite surface protein by IFA and a ∼60 kDa molecule in *P. falciparum* schizont lysate, as assessed by Western blot analysis [17], and induced **sterilizing immunity** in 2 out of the 10 monkeys being immunized in a first trial and 1 of the 8 monkeys immunized in a second trial [18]. This protection was associated with high specific binding activity to purified HLA-DRβ1*0401 molecules *in vitro* [18].

Being aware of the genetic control of the immune response, specially by MHCII molecules, and the exquisite specificity of the TCR-pMHCII complex formation in the induction of the appropriate immune response, modified HABP **24112** was inoculated in this study into in a new group of MHCII genotyped *Aotus* monkeys to analyze, at the structural level, the precise molecular mechanism of TCR/pMHCII complex formation resulting in induction of **sterilizing immunity**. For this purpose, spectratyping assays [19] were carried out to analyze the preferential usage of TCR β-chain variable region (Vβ) CDR3 families in those *Aotus* monkeys developing high antibody titers and **sterilizing immunity** against the *P. falciparum* parasite upon vaccination with the modified synthetic peptide **24112**, as well as in those that, despite developing lower antibody titers or not producing antibodies, were not protected.

The TCR–**24112**–MHCII complex associated with inducing **sterilizing immunity** against *P. falciparum* was studied by performing docking and energy minimization studies of this modified HABP with the previously reported X-ray crystallographic structure of the Hemagglutinin (HA) peptide, co-crystallized inside the HLA-DRβ1*0401–HA peptide–TCR complex [20], [21]. Care was taken to modify this reported structure according to the HLA-DRβ1*04 and TCR Vβ CDR3 amino acid sequence variations identified in protected and non-protected *Aotus* monkeys to determine spontaneous H bond formation, van der Waals (vdW) interactions, residue orientation and intermolecular distance differences between interacting atoms, to analyze this difference at the 3D structural level.

The results demonstrate, at the atomic level, that this first **sterilizing-immunity** **inducing** antimalarial peptide fits inside the specifically modified HLA-DRβ1*0403-**24112**–HA1.7 TCR complex, displaying a completely different pattern from that of monkeys carrying a different HLA-DRβ1*-like genotype that were not protected, thereby demonstrating that **immunogenicity** and induction of **sterilizing immunity** against transmissible diseases, like malaria, requires for **specifically modified** HABPs to fit appropriately inside the PBR of MHCII molecules so as to be specifically recognized by the TCR. Furthermore, the data here provided can be extrapolated to other molecules and pathogens as part of a logical and rational vaccine development methodology against transmissible diseases, malaria one of them.

## Results

### The 24112 modified peptide is highly immunogenic and induces sterilizing immunity in *Aotus* monkeys: the HLA-DRβ1*0403-like allelic variant

**Figure 1:**
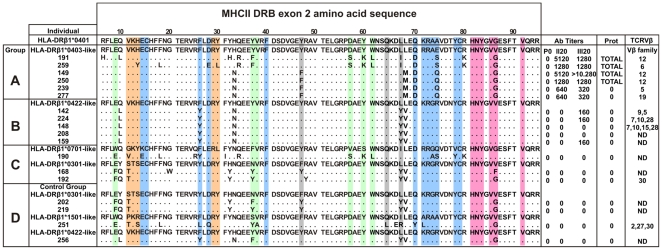
Genotyping, immune response and preferential TCR Vβ usage of immunized *Aotus* monkeys. Amino acid sequence of genotyped monkeys analyzed in this study. On top in the first row, the amino acid sequence of the reference HLA-DRβ1*0401 molecule as reported by Hennecke [20]. Monkeys were classified into different immunization groups (A, B, C and D) according to the similarity of their MHC class II DRβ exon 2 nucleotide sequences to HLA-DRβ1* alleles, displayed on top of each group as reference. Each monkey's antibody titers (Ab Titers) (determined by IFA) before (P0) or 20 days after each immunization (II20, III20), as well as protection results (Prot) against experimental challenge and expanded TCR βV families are shown. Residues conforming the HLA-DRβ1*04 pockets are highlighted in different colors according to the following code: pocket 1 (P1), pale fuchsia; pocket 4 (P4), blue; pocket 6 (P6), orange; pocket 7 (P7), gray and pocket 9 (P9), light green.

Based on the similarity of the MHC-DRB exon 2 of *Aotus* monkeys genotyped with HLA-DRβ1*alleles as described by Suarez et al. according to their peptide binding pockets [12], eighteen (18) *Aotus* were selected from a group of 40 nonfamily, non geographically-related genotyped *Aotus* monkeys and classified into 4 immunization groups as follows: Group A (HLA-DRβ1*0403-like) contained 6 MHC-AoDRβ*W45/47 monkeys which shared 92%–100% similarity with HLA-DRβ1*0403; Group B (HLA-DRβ1*0422-like) included 5 MHC-AoDRβ*06 typed monkeys showing 95% similarity to HLA-DRβ1*0422; Group C consisted of 2 MHC-AoDRβ1*03 (HLA-DRβ1*0701-like) monkeys sharing 89%–95% similarity with HLA-DRβ1*0301 and one MHC-AoDRβ*W38 (HLA-DRβ1*0701-like) monkey showing 87%–93% similarity to HLA-DRβ1*0701. An immunization control denoted as Group D contained 4 monkeys with diverse MHC-AoDRβ alleles due to the limited number of monkeys typing HLA-DRβ1*04-like [12] (**Figure 1**).

**Figure 2:**
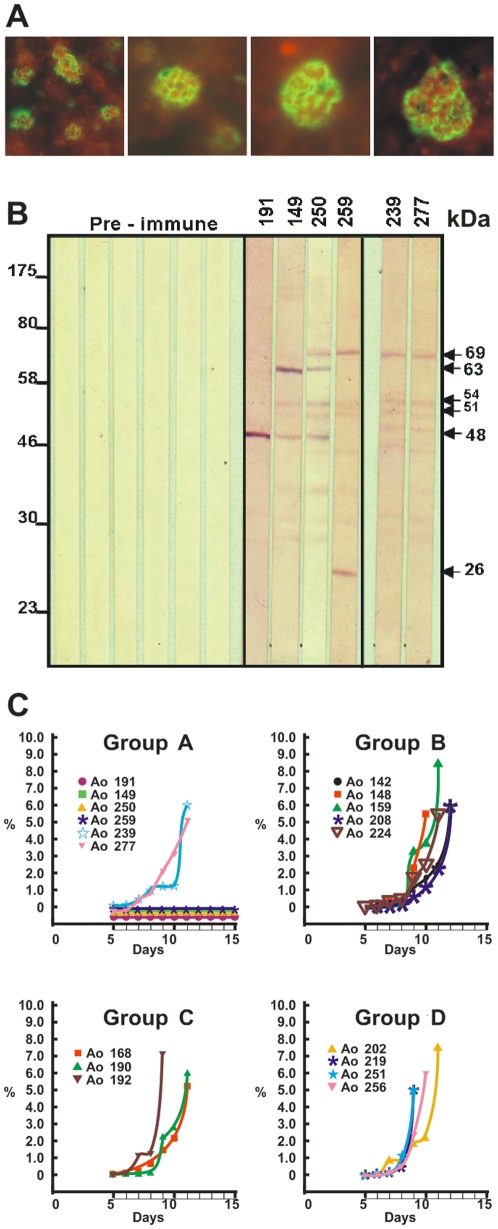
Immunological and protection studies. (**A**) Indirect immunofluorescence assay using group A *Aotus* monkey's sera (obtained 20 days after the 3^rd^ immunization with HABP **24112 and** 1 day prior to challenge), displaying a merozoite membrane reactivity pattern characteristic of MSP-2's localization in late schizonts. (**B**) Western blot analysis of schizont-lysate *P. falciparum* proteins reacting with sera from Group A monkeys (monkeys 192, 149, 250, 259, 239 and 277), obtained 20 days after the 3rd immunization and the day previous to the challenge, tested at 1∶200 dilution. Sera are recognizing *P. falciparum* proteins of 69 kDa and cleavage fragments of 63, 54, 51 and 48 kDa. (**C**) Parasitemias developed in immunized *Aotus* monkeys from groups A–C and the control group D. The percentage of parasitized red blood cells was evaluated daily by Acridine Orange staining read on a fluorescence microscope, starting on day 4 post-challenge and until day 15. In those **fully protected** *Aotus* monkeys the complete slide was read with ∼1.000.000 RBCs being screened. Each monkey in each group is represented by a different symbol.

Monkeys in Groups A, B and C developed two strikingly different immune responses after being vaccinated with peptide **24112** emulsified in Freund's adjuvant (**Figure 1**). Indirect immunofluorescence assays (IFA) detected very high antibody titers (≥1∶1,280) in four out of the six (∼67%) HLA-DRβ1*0403-like genotyped monkeys in Group A (Ao191, Ao250, Ao149 and Ao259) after the second and third immunizations, as indicated by the recognition of a merozoite membrane immunofluorescence pattern in late schizonts, which is in complete agreement with the surface localization of MSP-2 (**Figure 2A**). Furthermore, Western blot analyses of *P. falciparum*-schizont lysates using the same hyperimmune sera showed strong reactivity with protein bands of 63, 54, 51 and 48 kDa, which are close to the molecular weight of MSP-2 (∼69kDa) and its cleavage fragments. The remaining two HLA-DRβ1*0403-like monkeys in Group A (Ao239 and Ao277) developed lower antibody titers (1∶320) and showed a weaker Western blot recognition of MSP-2 and its cleavage fragments at the same serum dilution (1∶200) (**Figure 2B**).

While nonprotected control monkey developed very high parasitemias patent by day 5, reaching ≥5% by days 8–11 and requiring immediate treatment, the same four HLA-DRβ1*0403-like monkeys that developed high antibody titers (≥1∶1,280) were **fully protected** against experimental challenge. Protection being understood as the complete absence of blood parasites in the whole slide (screening under the fluorescence microscopy and with Acridine Orange staining of ∼1,000,000 RBCs per monkey per day) during the 15 days that the experiment lasted (**Figure 1** and **2C**). This indicated that **24112** confers **sterilizing immunity** to two thirds (66.6%) of the HLA-DRβ1*0403-like genotyped *Aotus* monkeys (Ao191, Ao250, Ao149 and Ao259). On the contrary, the other two monkeys (Ao239 and Ao277) producing lower antibody titers (1∶320) developed high parasitemia levels on day 4–5 post-challenge that were comparable to the levels shown by monkeys in the control group (**Figure 2C**).

Only three out of the five HLA-DRβ1*0422-like monkeys in Group B (Ao142, Ao224 and Ao159) developed low antibody titers (1∶160) after the third immunization, whereas no antibodies were detected in the other two Group B monkeys (Ao148 and Ao208). No anti-*P. falciparum* antibodies were detected in sera from Group C or Group D monkeys, as determined by IFA titers (**Figure 1**) and Western blot assays nor were any of the monkeys in Groups B, C or D protected against experimental challenge with *P. falciparum* (**Figure 2C**).

### Docking of peptide 24112 into the MHCII of protected and non-protected monkeys

Based on the strong association between the HLA-DRβ1*0403 genetic characteristic with **24112's** sterile immunity induction ability, the 3D structure of this modified HABP determined by us [17] and the 3D structure of the HLA-DRβ1*0401 molecule determined by Hennecke [20], [21], docking studies of **24112** were performed on this MHCII molecule.

**Figure 3:**
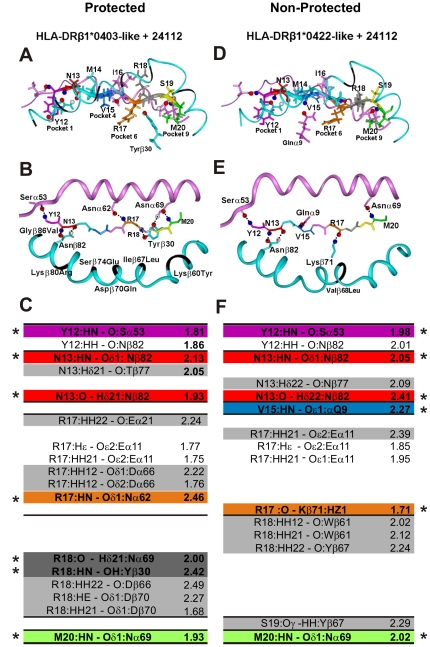
Interatomic interactions of peptide 24112 with HLA-DRβ1*04 molecules. Interaction of peptide **24112** with the HLA-DRβ1*0403-like of protected *Aotus* 191: (**A**) frontal view, (**B**) top view. Interaction of peptide **24112** with the HLA-DRβ1*0422-like complex of the non-protected *Aotus* 148: (**D**) frontal view, (**E**) top view. In the frontal view panels **A** and **D**, the orientation of **24112** residues' lateral-chains (represented as sticks) and their positions inside MHCII molecules are shown according to the following color code: Y12 (fuchsia in P1), N13 (red in P2), M14 (pale blue in P3), V15 (dark blue in P4), I16 (pink in P5), R17 (orange in P6), R18 (dark gray in P7), S19 (yellow in P8) and M20 (green in P9). Top view panels **B** and **E** display the H bonds (shown as doted lines) established between **backbone** atoms of peptide **24112** (represented as sticks) and **side-chain** atoms of residues from the MHCII α and β chains (depicted as pink and blue ribbons, respectively) in protected (group B monkeys) as well as non-protected (group E monkeys). The nitrogen and oxygen atoms are shown as blue and red balls, respectively. Black segments in the β-chain correspond to the residues that were modified according to the MHCII sequence of Ao191 (HLA-DRβ1*0403) and Ao148 (HLA-DRβ1*0422). (**C** and **F**) H bonds and vdW interactions, measured in Amstrongs (Å), between **24112** with HLA-DRβ1*0403 and HLA-DRβ1*0422 molecules. Interactions involving different atoms are highlighted in pale gray, while interactions involving common residues are not shadowed. The color code for those residues establishing such H bonds is the same used in Figure 1.

In our previous ^1^H NMR studies, peptide **24112** displayed a distorted type III′ β-turn spanning from residue Y3 to T6 and a classical type III′ β-turn structure between residues A11 and M14 [17], whereas the rest of the molecule was unstructured. Similar to the HA peptide's crystallographic structure complexed with the HLA-DRβ1*0401 molecule [20], **24112** peptide's residues fitting into Pocket 1 (Y12 fuchsia) and Pocket 9 (M20 green) were downwardly-orientated and deeply embedded into these pockets (**Figure 3A**), whereas residues fitting into Pocket 4 (V15 dark blue) and Pocket 6 (R17 brown) were more shallowly embedded than the above-mentioned residues [22], [23], [24].

Several other probable binding registers of **24112** were docked on HLA-DRβ1*0401 but none of them completely displayed the characteristic binding motives of this alleles [25] and none allowed a perfect fit inside the PBR of this MHCII molecule, therefore suggesting that the only probable structure was YNMVIRRSM (underlined residues fit into pockets 1, 4, 6 and 9, respectively).

Docking of **24112** into the HLA-DRβ1*0403 structure modified according to the MHCII sequence of protected Ao191 showed that the structure was stabilized by the spontaneous formation of SEVEN of the ten canonical H bonds [22], [23], [24] (**Figure 3B,C**). These **7 H bonds** formed between **24112** peptide's **backbone** atoms (written in one-letter code hereafter) and MHC lateral chain residues (written in 3-letter code throughout this manuscript) and their interatomic distances (shown in Amstrongs inside parenthesis, throughout this manuscript and indicted by an asterisk in **Figure 3C,F**) were established as follows: Y12 interacting with Pocket 1 Serα53 (in fuschia, 1.81 Å), N13 with **P2** Asnβ82 (in red, 2.13 Å), N13 with **P2** Asnβ82 (in red, 1.93 Å), R17 with Pocket 6 Asnα62 (in brown, 2.46 Å); R18 with Pocket 7 Asnα69 (in dark gray, 2.0 Å), R18 with Pocket 7 Tyrβ30 (in dark gray, 2.42 Å) and M20 with Pocket 9 Asnα69 (in green, 1.93 Å).

By the same token, docking of **24112** into the modified HLA-DRβ1*0422 structure of Ao148 (non-protected, non-antibody producer monkey) showed the spontaneous formation of SIX of the 10 canonical H bonds between atoms of the peptide's **backbone** and MHCII lateral chain residues (**Figure 3E,F**). Binding of Y12, N13 and M20 involved the same residues mediating binding of **24112** to HLA-DRβ1*0403 in Ao191, but displayed a totally different H-bonding pattern in the **24112**–HLA-DRβ1*0422 complex between V15 with Pocket 4 Glnα9 (in dark blue, 2.27 Å), and R17 with Pocket 6 Lysβ71 (in brown, 1.71 Å). It can be clearly seen that **no H bonds** are established between **24112**'s R18 backbone and lateral chains of HLA-DRβ1*0422 At this point, it is worth to remember that H bonds are not determined by their interactions distances but also by their torsion angles.

Additional differences were observed between these two pMHCII complexes (highlighted in pale gray in **Figure 3C,F**). In the HLA-DRβ1*0403 molecule, vdW interactions were preferentially established between **lateral chain** atoms of N13 with Thrβ77, R17 with Gluα21, Gluα11 and Aspα66 (the latter two confer a negatively charge character to pocket 6 that strongly stabilizes binding of positively charged residues like R, K or H [23]), a polarity further accentuated by the Aspβ70Gln difference in this allelic variant. H bonds are also established between lateral chain atoms of R18 with Aspβ66 and Aspβ70.

Meanwhile in HLA-DRβ1*0422 peptide **24112**, vdW interactions (**Figure 3F**, pale gray) are specifically established between lateral chain atoms of N13 with Asnβ77, R17 with Gluα11, R18 with Trpβ61 and Tyrβ67, and S19 with Tyrβ67; displaying a strikingly different H bonding and vdW interaction pattern in Pocket 6, that results in a different orientation of R18 (**Figure 3D**). These H-bonding differences approximate R18 to Trpβ61 in the HLA-DRβ1*0403-like–**24112** complex preventing an appropriate H bonding between backbone atoms of **24112** with Asnα69 and Tyrβ30 (**Figure 3A**) residues of the HLA-DRβ1*0422 molecule, as situation clearly visible in **Figure 3D**, were these two H bonds that are critical for the anchoring and stabilization of the peptide inside the PBR are not observed in **Figure 3E** nor measured in **Figure 3F**. It is well know that H bonds give high stability and specificity to the pMHCII complex formation due to the exquisite energetic dependence on the stereochemistry and geometry of the bond.

### Preferential usage of Vβ families in immunized monkeys

The spectratyping analysis of the TCR repertoire provides a global picture based on a reduced number of cells, and helps determining the complexity and stability of a T cell repertoire in response to an antigenic stimulus, but it only provides a qualitative description of the T-cell mediated immune response raised against a particular antigen. Nevertheless, the methodology here reported is useful, specific and sensitive enough for detecting the different TCR CDR3β sequences that expand in response to a specific antigen.

Based on the *Aotus* Vβ sequences reported to date [10], [11], the spectratyping analysis of the CDR3 amplicons showed a polyclonal Gaussian-like distribution for the majority of TCR Vβ families in the **pre-immune** (P0) samples (**Figure S1a)** typical of unstimulated T cells. The CDR3 size distribution pattern (**Figure S1b**) changed strikingly to skewed oligoclonal expansions of one, two or three dominant Vβ families in Ao191, Ao259, Ao149, Ao250, Ao239, Ao277, Ao142, Ao224 and Ao148 upon immunization with peptide **24112**; however, such skewed patterns were only associated with high antibody production in Ao191, Ao259, Ao149, Ao250, Ao239 and Ao277. CDR3 Vβ distribution of control monkeys receiving only Freund's adjuvant (Group D) showed Gaussian-like patterns similar to the ones found in P0 samples.

Post-immunization lymphocyte samples of the fully-protected HLA-DRβ1*0403-like genotyped monkeys (Group A) developing high antibody titers after the second (II_20_) and third (III_20_) doses showed a selectively higher usage of the TCR families Vβ12 (Ao191, Ao149 and Ao250) and Vβ6 (Ao259) in T-cell clones expanding in response to immunization with **24112** (**Table S1**). Striking differences were found in the TCR family usage of the non-protected monkeys Ao239 and Ao277 developing lower antibody titers against *P. falciparum*, which preferentially expressed Vβ5 and Vβ19 TCR families (**Table 1**).

**Table 1:** TCR β-chain VDJ amino acid sequences expanded in response to immunization with peptide 24112.

Individual	TCRVβ Family	Vβ^a^ sequence	nDn^b^	Jβ^c^ sequence	TCRJβ Family	HLA-DRβ1
		92		111		
Human Influenza	Vβ3S1	LESASTNQTSMYL***CASS***	STGLP	YGYT***FGSG***TRLTVVEDLNKVFPPEVAVF	1.2	0401
**Antibody producer and protected individuals**						
Ao191•	Vβ12-3	IQPSEPGDSAVYF***CASS***	FLEGG	YDYT***FGSG***TRLTVVDDLSKVFPPTVAVF	1.2	0403
Ao250	Vβ12-1	IQPSEPRDSALYL***CASS***	QQG	NYDYT***FGSG***TRLTVVDDLSKVFPPTVAVF	1.2	0403
Ao259	Vβ6-10	LEAAAPSQTSVYF***CASS***	DLLSA	NYDYT***FGSG***TRLTVVDDLSKVFPPTVAVF	1.2	0403
Ao149	Vβ12-6	IQPSEPRDSAVYF***CASS***	LASGS	TDPLY***FGPG***TRLTVLDDLNKVFPPTVTVA	2.3	0403
**Antibody producers and non-protected individuals**						
Ao239	Vβ5-4	LSSLELGDSALYF***CASS***	VGIRD	YDYT***FGSG***TRLTVVDDLSKVFPPTVAVF	1.2	0403
Ao277	Vβ19-12	LTSAQWNQTAFYL***CASS***	TPI−−	NYDYT***FGSG***TRLTVVDDLSKVFPPTVAVF	1.2	0403
**Non-antibody producers or protected individuals**						
Ao148•	Vβ15-5	IRSSGLGDAATYL***CASS***	RDEED	YDYT***FGSG***TRLTVVDDLSKVFPPTVAVF	1.2	0422
Ao148	Vβ28-9	STNQTSVYL***CASS***	LYLRG	NYDYT***FGSG***TRLTVVDDLSKVFPPTVAVF	1.2	0422
Ao224	Vβ28-9	STNQTSLYL***CAS***	FAGDI	EAF***FGEG***TKLTVVDDLSKVFPPTVAVF	1.1	0422
Ao142	Vβ9-9	VSGLELGDSALYL***CAS***	NLVLWTGVR	NYDYT***FGSG***TRLTVVDDLSKVFPPTVAVF	1.2	0422
Ao148	Vβ7-6	IQRTKQGDSAMYL***CASS***	PSGAGS	TDPLY***FGPG***TRLTVLDDLNKVFPPTVAVF	2.3	0422
Ao224	Vβ7-1	IQRTKQGDSAMYL***CASS***	SDGLY	TDPLY***FGPG***TRLTVLDDLNKVFPPTVAVF	2.3	0422
Ao142	Vβ5-3	VSGLELGDSALYL***CAS***	NTGLADCPC	TDPLY***FGPG***TRLTVLDDLNKVFPPTVAVF	2.3	0422

CDR3 sequences are limited by ^92^CASS and FGXG^111^ motifs (in bold and italic).

**^a^Vβ:** β-chain variable region amino acid sequences.

**^b^nDn:** non-templated diversity-n region.

**^c^Jβ:** β-chain diversity joint region families. Big black dots correspond to the *Aotus* Vβ amino acid sequences used to replace the corresponding residues in the V3S sequence of the HLA-DRβ1*0401–HA–HA 1.7 TCR complex, numbered according to the crystallographic structure reported by Hennecke [20] and shown in the first row.

HLA-DRβ1*0422-like non-protected monkeys (Group B) developing low or no detectable antibody titers (**Figure 1**) showed a preferential usage of Vβ9 and Vβ5 in Ao142; Vβ7, Vβ10 and Vβ28 in Ao224; and Vβ7, Vβ15 and V28 in Ao148 (**Table S1**), but none of them displayed a preferential usage of Vβ12 and Vβ6 families, therefore suggesting an exclusion mechanism at the TCR level between protected and nonprotected monkeys in the TCR Vβ usage, in spite that some of them can produce antibodies (although at a lower level) when being immunized with the same epitope. Therefore these later antibodies must have different affinities and/or structural characteristics, a subject currently under study at our institute.

### Sequencing of TCR-Vβ CDR3 segments

The Vβ CDR3 sequences of expanded T-cell clones indicated a random usage of J segments in each of the expanded Vβ families. For example, Vβ12 CDR3 expanded sequences of the antibody-producer protected Ao191 and Ao149 monkeys included almost all possible J segments (J1.1, J1.2, J1.4, J2.1, J2.2 and J2.3; **Table S1**). This shows that different T lymphocyte subpopulations with similar CDR3β lengths are involved in the **sterilizing immune response** induced by peptide **24112** and suggests a clear polyclonal response to a molecularly defined antigen in individuals with specific and determined genetic backgrounds, clearly visible in the reactivity of these sera in Western blot analyses (**Figure 2B**).

The amino acid sequence variations found in the Vβ12 D region of the fully protected Ao191 (**Table 1**; clone 3) as well as the differences detected in the Vβ15 D region of the non-protected, non-antibody producer Ao148 (**Table 1**; clone 5) were used to modify the Vβ3S1 sequence (belonging to the same Vβ12 family) of the HLA-DRβ1*0401–HA–HA1.7 TCR crystal structure [20], [21], as described in detail below.

### Structural analysis of the MHCII–24112–TCR complex

The previously reported HLA-DRβ1*0401–HA–HA1.7 TCR crystal structure [20], [21] was modified to determine the HLA-DRβ1*0403-like–**24112**–TCR structure of the fully protected Ao191 and the HLA-DRβ1*0422-like–**24112**–TCR structure of the non-protected Ao148. These molecules were analyzed and compared, at the structural level, to examine H bond formation and distance differences. For the HLA-DRβ1*0403-like–**24112**–TCR complex, the TCR Vβ3S1 sequence was modified in the CDR3β region according to the variations found in the Vβ12 D region of clone 3 from protected Ao191 as follows (TCR residues are written in three-letter code throughout this manuscript): Phe3β96Ser, Leu3β97Thr, Glu3β98Gly, Gly3β99Leu, Gly3β100Pro and Asp3β105Gly in the J region (**Table 1** and **Table S1**), which are numbered according to Hennecke's system [21]. For the HLA-DRβ1*0422-like–**24112**–TCR complex, the TCR Vβ3S1 was replaced by the Vβ15 family sequence found in clone 5 from the non-protected, non-antibody producer Ao148 as follows: Arg3β96Ser, Asp3β97Thr; Glu3β98Gly; Glu3β99Leu; Asp3β100Pro and Asp3β105Gly in the J region.

When the complete HLA-DR1*0403–**24112**–TCRVβ12 modified complex was superimposed onto the original 3D structure of the HLA-DRβ1*0401–HA–HA1.7 TCR complex [20], [21], it displayed a 1.41 r.m.s.d. Similarly, the HLA-DRβ1*0422–**24112**–TCRVβ15 modified complex displayed a 1.32 r.m.s.d. when it was superimposed with HLA-DRβ1*0401–HA–HA1.7TCR complex.

**Figure 4 pone-0009771-g004:**
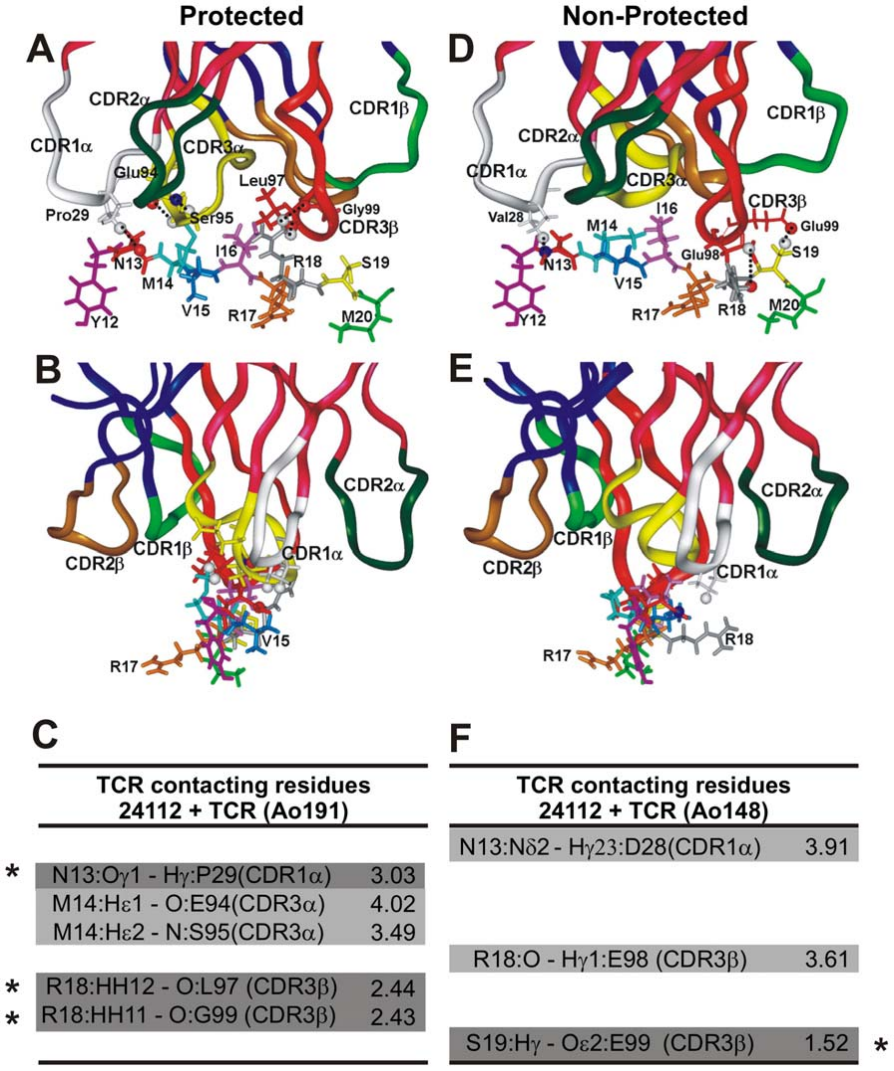
Peptide 24112 interactions with modified HA 1.7 TCR. (**A,B**) H bonds and vdW interactions established between peptide **24112** with the HA 1.7 TCR molecule modified according to the Vβ12 clone 3 sequence of protected *Aotus* 191. (**D,E**) H bonds and vdW interactions of the peptide **24112** with the HA 1.7 TCR molecule carrying the same Vβ12 family in the Vβ15 clone, modified according to Vβ15 clone 5 sequence of the non-antibody producer, non-protected *Aotus* monkeys 148. Small black dots correspond to peptide **24112**'s atoms making contact with modified HA 1.7 TCR molecule atoms. Amino acids conforming each TCR CDR are: in the TCR α-chain (dark pink ribbons shown on top): Tyr24 to Tyr31 for CDR1α (white ribbon), Lys48 to Leu55 for CDR2α (dark green ribbon), and Ser93 to Leu104 for CDR3α (yellow ribbon); in the TCR β-chain CDR (dark blue ribbons on top) residues were: Val24 to Asn31 for CDR1β (light green ribbon), Phe48 to Glu56 for the CDR2β (gold ribbon), and Ala93 to Gly109 for CDR3β (red ribbon) [2], [3], [20]. (**B,E**) Frontal view of the same complex and view rotated 90°. (**C**,**F**) Same H bonds (dark gray) and vdW interactions established between peptide **24112** with Ao191-Vβ12 TCR (left column) and Ao148-Vβ15 TCR (right column), respectively, with their corresponding interatomic distances indicated in Amstrongs (Å). Interactions involving different atoms are highlighted in pale gray, whereas interactions involving the same residues in both complex are not highlighted.

Based on the above mentioned information and the fact that TCR Vα displays limited polymorphism (therefore their CDRs were not cloned in this study), the 3D structure of the HLA-DRβ1*0401–HA–HA1.7 TCR complex, used as template, showed in docking analyses that binding of such modified HA1.7 TCR to both HLA-DRβ1*04–**24112** complexes is mediated by the interaction of the TCR Vα region with the peptide's N-terminal portion, while the C-terminal fragment established contact with Vβ regions (**Figure 4A,D**), as previously described elsewhere [20], [21], [26].

### 24112 atoms recognized by modified TCR CDRs

While **24112** is deeply buried inside the HLA-DRβ1*0403 molecule (**Figure 3A**), N13 (corresponding to **P2** in red), M14 (**P3** in pale blue), I16 (**P5** in pink), R18 (**P7** in gray), and S19 (**P8** in yellow) are upwardly-oriented, particularly R18 (gray), and therefore available to TCR inspection (**Figure 4A,B**). The CDR3β Vβ12 of protected Ao191 shows spontaneous formation of THREE H bonds (shown as small dots in **Figure 4A,B**; highlighted in dark gray in **Figure 4C** where they are indicated by asterisks): one between N13 with Proα29 from CDR1α (white ribbon on top), two between R18 with Leuβ97 (2.44 Å) and R18 with Glyβ99 (2.43 Å) from CDR3β (red ribbon on top). TWO more vdW interactions are observed between M14 with Gluβ94 (4.02 Å) from CDR3α (yellow ribbon), and M14 with Serα95 from CDR3α (yellow ribbon on top) (3.49 Å).

In the analysis of the TCR contacting residues of HLA-DRβ1*0422–**24112** complexed with the CDR3β Vβ15 region of the non-protected Ao148 (**Figure 4** **D,E**,**F**) only ONE H bond (**Figure 4F**, dark gray indicated by an asterisk) is spontaneously formed between S19 and Gluβ99 (1.52 Å), while TWO vdW interactions are observed between N13 with Aspα28 (3.91 Å) from CDR1α (in white ribbon), and R18 with Gluβ98 (3.61 Å) from CDR3β (in red ribbon), perhaps due to the horizontal localization of this peptide's residue R18 (**Figure 4E**). This complex displays therefore fewer and weaker electrostatic interactions with the TCR.

### TCR footprint on the HLA-DRβ1*04 complexed molecules

**Figure 5:**
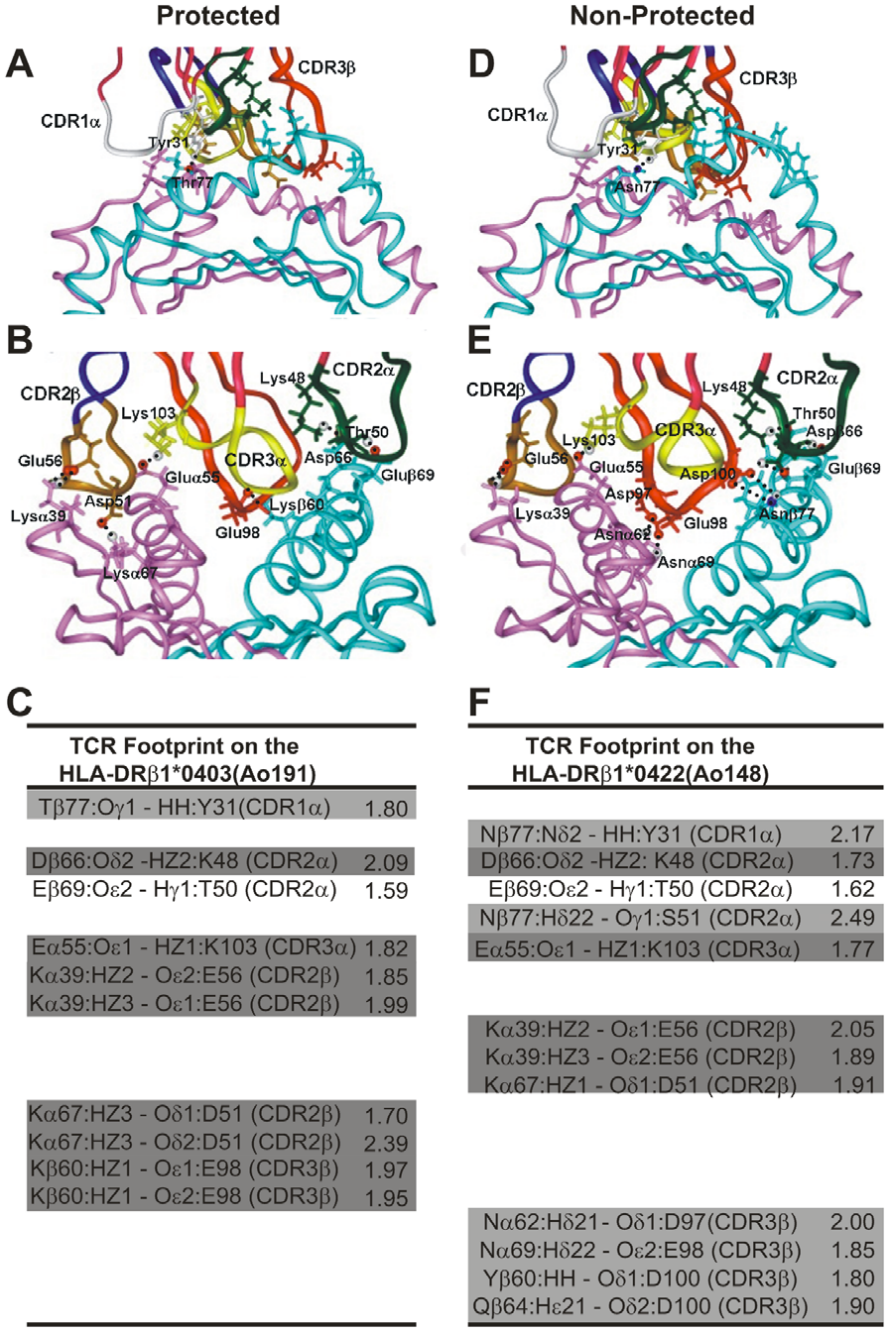
Footprint of modified HA 1.7 TCR on HLA-DRβ1*04 molecules. (**A,B**) Footprint of the HA 1.7 TCR molecule modified according to the Vβ12 clone 3 sequence of protected *Aotus* 191, in HLA-DRβ1*0403 molecule. (**D,E**) Footprint of the HA 1.7 TCR molecule modified according to Vβ15 clone 5 sequence of the non-antibody producer, non-protected *Aotus* monkeys 148 on the HLA-DRβ1*0422 molecule. (**B,E**) Frontal views of the same complexes and view rotated 90°. (**C,F**) H bonds established between HLA-DRβ1*0403 and HLA-DRβ1*0422 with HA1.7 TCR-like modified molecules with their corresponding interatomic distances indicated in Amstrongs (Å). Salt bridges are highlighted in dark gray while interactions involving different atoms are highlighted in pale gray, and interactions involving the same residues in both complexes are not highlighted.

**Figure 5** shows the H bonds established between the HLA-DRβ1*0403-modified molecule and the Vβ12-clone-3-modified TCR (left) of the antibody-producer, protected Ao191 with their corresponding interatomic distances (**Figure 5** **, A,B** and **C** left panels), as well as the 3D structure and interatomic distances between the HLA-DRβ1*0422-modified molecule and the Vβ15-clone-5-derived TCR of the non-antibody producer, non-protected Ao148 (**Figure 5D, E, and F** right panels).

Taking the human influenza TCR–pMHCII complex as a template, the footprint analysis of these TCRs on their corresponding MHCII molecules shows that in HLA-DRβ1*0403 H bonds are specifically established with CDR1α (**Figure 5D**, white ribbon) residues (Thrβ77 with Tyr1α31), CDR2β (**Figure 5A**, brown ribbon) residues (Lysα39 with Glu2β56, Lysα67 with Asp2β51), and CDR3β (**Figure 5A**, red ribbon) residues (Lysβ60 with Glu3β98) [21], [27], [28], [29]. HLA-DRβ1*0422 preferred specific interactions between CDR1α (**Figure 5A**, white ribbon) residues (Asnβ77 with Tyr1α31) and CDR2α (**Figure 5E**, green ribbon) residues (Asnβ77 with Ser2α51), CDR2β (**Figure 5E**, brown ribbon) residues (Lysα39 with Glu2β56 and Asp2β51), and CDR3β (**Figure 5E**, red ribbon) residues (Asnα62 with Asp3β97, Asnα69 with Glu3β98, Tyrβ60 with Asp3β100, and Glnβ64 also with Asp3β100), which induced a **twist** and slight displacement towards HLA-DRβ1*0422 α-chain residues not quantitatively determined in this study, but clearly seen in **Figure 5D and E**. The last three contacts have not been described among the canonical **evolutionarily conserved** **amino acids** that control TCR–MHC interactions, suggesting a different TCR contacting mode in this non-protective complex [27], [28], [29], [30], [31].

The distances and electrostatic properties also show that only five (5) **salt bridges** (**Figure 5C,F**; dark gray) are established between the residues conforming the non-protective HLA-DRβ1*0422–Vβ15 TCR complex, whereas in the protection-associated HLA-DRβ1*0403–Vβ12 TCR complex, eight (8) **salt bridges**, are determined suggesting a stronger and more stable interaction of this pMHCII complex; a condition needed to stabilize the complex and properly activate the immune system toward a **sterilizing immunity** response.

It has been clearly shown that the strongest interactions are established between lateral chain atoms of MHCII molecules and atoms from the **peptide's backbone** (**Figure 3**), whereas in the **peptide/TCR** interaction the strongest bonds are established with the peptide's lateral chain atoms (**Figure 4**), among which salt bridges are the most important electrostatic forces.

In essence, this study clearly shows that important differences exist between residues' orientation and electrostatic forces established in these TCR/pMHCII complexes, which are associated with two different immunological outcomes induced by the same peptide in slightly different variants of the same HLA-DRβ1*04 allele.

## Discussion

The structural data obtained in this study at the 3-dimensional level provides strong evidence that the TCR/pMHCII complex formed with the specifically modified HABP **24112** derived from the *P. falciparum* MSP-2 protein, inductor of high anti-parasite antibody titers and **sterile immunity** in 67% of HLA-DRβ1*0403-like genotyped *Aotus* monkeys (**Figure 1**), has a strikingly different 3D structure conformation from the one observed in non-protected non-antibody producer HLA-DRβ1*0422-like genotyped monkeys immunized with the same peptide (**Figures 3** **–** [Fig pone-0009771-g004] **5**). This is clearly demonstrated by a large set of differences in the salt bridges, H bonds and vdW interactions, interatomic distances and contacting residues' orientation between both complexes.

In this study, we show that *Aotus* monkeys developing high antibody titers and being protected against experimental challenge with the lethal *P. falciparum* malaria FVO strain carry the HLA-DRβ1*0403-like genetic marker and have a preferential usage of Vβ12 and Vβ6 TCR families (**Table 1**), whereas the same molecule was poorly or not immunogenic nor protection-inducing in *Aotus* monkeys carrying a slightly different HLA-DRβ1*04 allelic variant like HLA-DRβ1*0422 and that such weaker and/or nonprotective immune response was associated with the preferential usage of TCR Vβ 9, 5, 7, 10, 15 and 28 families; therefore confirming the exquisite specificity of the TCR/pMHCII formation in **sterilizing immunity induction** against malaria.

While Group C monkeys immunized with **24112** and carrying some other non HLA-DRβ1*04-like alleles did not develop any detectable antibody titers nor were protected against experimental challenge, same as monkeys in control Group D (inoculated with Freund's adjuvant only), these data show that peptide **24112** is neither recognized nor presented by these MHCII alleles, a fact corroborated by the inability of **24112** to bind experimentally to other purified HLA-DRβ1^*^ molecules (Group C) [17], [18]. Therefore the immune response observed in Group A HLA-DRβ1*0403 monkeys is HLA-DRβ1*0403-specific and not the result of an unspecific stimulation induced by the Freund's adjuvant, as show by Group D results (here only one HLA-DRβ1*0422 monkey was included due to the low frequency of this allelic variant).

Docking studies clearly demonstrate that the TCR/pMHCII complex has to be properly assembled in order to induce **sterilizing immunity** against this deadly disease, expanding and confirming the elegant structural studies on **antigenicity** with the Influenza virus Hemagglutinin A (HA) peptide [22], **immunogenicity** with several immunodominant epitopes from hen egg lisozyme (HEL) [32] and Conalbumin (CA) [33], **autoimmunity** induced by the Col II peptide in rheumatoid arthritis [24] and many more. However it should be highlighted that the present study is hitherto the first immunogenicity and **sterilizing immunity** study on against any microbe thoroughly analyzed up to the structural level.

Bearing in mind that the **24112** modified peptide induced **high immunogenicity** and **sterilizing immunity** in 67% of HLA-DRβ1*0403-like genotyped monkeys, these data clearly suggest that **additional** modified HABPs should be included in a vaccine formulation in order to activate more TCR/pMHCII complexes and to develop **complete protection** against malaria in HLA-DRβ1*0403 individuals. Furthermore, a substantially larger number of modified HABPs has to be included in a fully effective, multiepitopic, multistage, minimal subunit-based, chemically synthesized antimalarial vaccine capable of conferring protection to ALL different HLA-DRβ1* alleles, and variants, even more considering that the parasite employs multiple molecules and invasion mechanisms [34], the majority of which display a large number of exquisite genetic variations to evade the host's immune system pressure that are able to distract the complete immune system b y displaying just one amino acid variation [35].

Therefore all parasite pathways and strategies for invading RBCs and liver cells (or at least the most relevant ones), must be destroyed or at least blocked to prevent parasite's entry into target cells. This notion is further supported by the recent transcriptome analyses demonstrating that ∼58–90 of all *P. falciparum* proteins are involved directly in RBC invasion [36] and probably a similar number are involved in sporozoite invasion to the liver cells [37].

In our docked structures of the protection-associated HLA-DRβ1*0403-**24112**–TCR complex, the most variable regions of the TCR (CDR3α (yellow) and CDR3 β (red)) are located in the central portion of the binding interface with **24112,** while the most conserved CDR1 and CDR2 regions make contact with the upper surface of the MHCII helices that are surrounding CDR3 like a gasket [30], [31]. Although residues M14 in **P3** (pale blue) and I16 in **P5** (pink) are not establishing H bonds or vdW interactions with the TCR, they fit perfectly well inside the hydrophobic grooves.

Meanwhile, the CDR3α does not make contact with any **24112** contacting residues (e.g. **P3** (M14), **P5** (I16), **P7** (R18)) in the **24112**–HLA-DRβ1*0422 complex, and fewer H bonds and vdW forces are established in this complex, which suggests little probability of TCR inspection and weaker interaction of this pMHCII complex with the TCR in this non-protective complex.

The striking variations found between the Vβ15 clone-5 TCR–**24112**–HLA-DRβ1*0422 and the Vβ12-clone-3 TCR–**24112**–HLA-DRβ1*0403 complexes in the CDRs usage of **evolutionary conserved residue** contacts [27], [28], [29], [30], [31], specifically regarding salt bridge formation (5 in the former complex and 8 in the later complex) as well as in the orientation of contacting residues in the TCR-MHCII footprint, suggests a different recognition pattern and a different docking footprint on the TCR/pMHCII interaction between these 2 complexes associated with two different immune responses: non-antibody production and non-protection versus antibody production and protection induction, respectively.

The conservation of the critical residues controlling the “TCR/pMHCII interaction” suggests that the vast peptide's recognition specificity displayed in **sterilizing** **immunity** against modified peptides is almost entirely driven by the appropriate configuration of the HLA-DRβ1*0403–**24112** peptide complex, which displays a completely different array of H bonds and vdW networks compared to the non-immunogenic, non-protective HLA-DRβ1*0422–**24112** peptide complex structure [27], [28], [29], [30], [31].

Studies by Kersh et al. [38] on the binding of Hemoglobin (Hb 64–76) peptide to I-E^K^ molecules have elegantly shown that subtle modifications such as replacing the D residue in the E73D sequence of the Hb peptide fitting inside Pocket 6 modifies the orientations and interatomic distances of **P5**, **P7** and **P8** with their TCR contacts, which is in turn reflected in a 1,000-fold reduction in the potency of the altered peptide **24112** to induce antibody production. Our situation is different since the same peptide is anchored to both TCR/pMHCII complexes but the slight differences within the genetic background of the *Aotus* HLA-DRβ1*04-like molecules lead to different H bonding and vdW patterns, specially between the peptide's backbone atoms and lateral chains residues of the pMHCII molecule. Furthermore, conserved residues controlling the “TCR/pMHCII interaction” display a different hydrogen bonding and salt bridge formation pattern.

Since in previous studies in which we had characterized the *Aotus* MHC-DRβ exon 2 [12], [39] in a large number of monkeys show that the amino acids that define the PBR **pockets** share a mean similarity of 89–94% with HLA-DRβ1*0403 (with a minimum similarity of 82–88% and a maximum of 94–100%), a mean similarity of 91% with HLA-DRβ1*0422 (a minimum similarity of 88% and maximum of 94%), and quite similar similarity values with the other alleles analyzed in this study. Furthermore since most substitutions are functionally and evolutionary conserved [40], [41], we can conclude that the results observed in the *Aotus* monkey model mimic in a very high degree the TCR-pMHCII complex formation in humans, thus highlighting the extreme importance of this nonhuman primate for human vaccine development.

Given that Glnα9, Asnα62, Asnα69, Trpβ61 and Asnβ82 residues establishing H bonds with the peptide's backbone are conserved among humans and *Aotus* (unpublished results), these data suggest that the modified HABP **24112** properly fitting into HLA-DRβ1*0403 molecules **could be used almost immediately for human vaccination** in individuals carrying this allelic variant and highlights this peptide as the **first component** of a **sterilizing-immunity-inducing,** multiepitopic, multistage, minimal subunit-based, chemically synthesized **antimalarial vaccine for human use**.

Furthermore, the data clearly show that developing fully effective vaccines is a far more elaborated and complex process than merely vaccinating humans with recombinant fragments (either individually or as mixtures), viral vector, DNA-based or unmodified short or long synthetic peptides [42], which have lead to «disappointing» and frustrating results [42], [43], [44], some of them with deleterious effects. The evidence also indicates that the molecular characteristics of the microbe and the host play a fundamental role in the conformation of the appropriate TCR/pMHCII complex to induce **sterilizing immunity.**

Finally, it is worth noting that all the evidence shown here was obtained by using the most stringent system to asses **sterilizing immunity** (since a 100% infective dose of an *Aotus*-adapted *P. falciparum* strain was **intravenously** inoculated into monkeys), which allow us to conclude that **multiantigenic**, **multistage**, **minimal subunit-based**, **specifically-modified**, **chemically-synthesized vaccines** against any transmissible disease like malaria, must be **appropriately designed** and modified to fit perfectly well insidethe TCR/pMHCII complex so as to induce **sterilizing immune responses.**

## Materials and Methods

### Peptides

**24112** monomer and polymers were synthesized by using *t*-boc chemistry [45]. Polymerization was allowed by adding CG residues to the N and C terminal ends of peptide **24112** (^24^SK**YS**NTF**NI**NA**Y**NMV**I**RRSM^43^), with a carefully standardized oxidation procedure that guarantees the formation of high molecular weight polymers (8–24kDa) for immunization purposes.

### *Aotus* monkeys

Forty wild-caught *Aotus* monkeys were kept in stainless-steel cages at FIDIC's primate station in Leticia, Amazonas, Colombia, and maintained in strict accordance with the NIH guidelines for animal care and the Colombian Ministry of Health (Law 84/1989), under the weekly supervision of CORPOAMAZONIA officials and a primatologist. Monkey sera (1∶20 dilution) were screened by IFA to determine previous exposure to *Plasmodium* parasites. Monkeys testing positive were returned to the jungle without further manipulation [8]. Monkeys' parasitemias were assessed daily by reading under fluorescence microscope by screening 1.000.000 Acridine Orange stained RBCs and they were immediately treated whenever *P. falciparum* infected RBCs were ≥5%, or before if the monkey's health condition had deteriorated. Treatment consisted of orally administered pediatric doses of Chloroquine (10 mg/kg on the first day and 7.5 mg/kg per day until day five). Once assuring total clearance of parasites from blood and excellent health condition, monkeys were released back into their natural habitat close to the site where they had been captured with the supervision of CORPOAMAZONIA officials. All procedures were approved and supervised by FIDIC's Ethics Committee in Health Research (Resolution No. 008430 of 1993, Colombian Ministry of Health) and by FIDIC's Primate Station Ethics Committee.

### MHCII-DRB genotyping

Genomic DNA of each monkey was isolated from peripheral blood lymphocytes to amplify the MHCII-DRβ exon 2 segment using high-fidelity PCR (ACCUZYME DNA Polymerase), as described elsewhere [12]. Sequences were obtained by cloning amplicons into the pCR®Blunt vector (Invitrogen™) and allele types were assigned by comparing them to previously reported *Aotus* alleles [12].

### Immunization and challenge

Based on their similarity to human HLA-DRβ1* alleles determined by genotyping [12], eighteen monkeys were selected for this study and classified into different immunization groups (**Figure 1**). Groups A (6 monkeys), B (5 monkeys) and C (3 monkeys) were immunized on days 0, 20 and 40 with 125 µg of peptide **24112** emulsified in Freund's complete adjuvant for the first dose, and in Freund's incomplete adjuvant for the second and third doses. Group D (4 monkeys) received only Freund's adjuvant on the same days. One milliliter of peripheral blood was collected on day 0 (pre-immune or P0) and 20 days after the second and third immunizations (II_20_ and III_20_, respectively) to obtain lymphocytes and sera for immunological studies. On day 60, all monkeys were challenged by **intravenous** inoculation of a 100% infective dose (100,000 infected erythrocytes) of the *P. falciparum* FVO *Aotus*-adapted strain, freshly obtained from a previously infected monkey. Blood parasitemia levels were monitored daily for 15 days by Acridine Orange staining. **Sterilizing immunity** was defined as the **complete absence** of parasites in the blood of protected monkeys during the 15 days that the experiment lasted, as assessed by reading the whole slide (∼1.000.000 RBCs were screened). Controls and non-protected monkeys developed patent parasitemias by day 5 and high parasitemias (≥5%) by days 8–11 after the challenge, therefore requiring immediate treatment (**Figure 1C**). Monkeys were kept in quarantine and released back into the jungle in company of CORPOAMAZONIA officials.

### IFA antibody titers

Air-dried slides containing *P. falciparum* late-schizonts (FCB-2 strain) obtained from a synchronized continuous culture were used for determining antibody titers, using twofold serial dilutions of monkey sera (initial dilution: 1∶40). Slides were washed 6 times with PBS, incubated with the appropriate dilution of affinity purified FITC-labeled goat anti-*Aotus* F(ab')2 IgG fragment, washed and read by fluorescence microscopy.

### Western blot analysis

Briefly, whole schizonts' lysate of the same FCB-2 synchronous culture was separated in a discontinuous PAGE system, electrotransfered to nitrocellulose membranes and incubated with appropriate monkeys' sera dilutions and with goat anti-*Aotus* IgG F(ab')2 fragment conjugated to Alkaline phosphatase, to asses immunoreactivity.

No attempts were made to determine cellular immune responses due to the limited amount of drowned blood and the priority given to the T cell cloning and spectratyping analyses.

### Characterization of the TCR repertoire

The cDNA was synthesized with the SuperScript™ III kit (Invitrogen, CA USA) using RNA isolated from peripheral blood lymphocytes as template. Nineteen specific forward primers and a common primer annealing in the β-chain constant (Cβ) region were designed to amplify all *Aotus* Vβ families reported to date [11]. Additionally, a set of reverse and forward primers amplifying a 419-bp fragment of the *Aotus* TCR α-chain constant (Cα) segment was used as amplification control (**Table S2**).

The Cα segments were coamplified together with each of the 19 CDR3 Vβ segments using 0.25 µM of Cα forward and reverse primers, and 0.75 µM of Vβ and Cβ primers. Amplification products were subjected to 6 run-off reaction cycles using Cα and Cβ reverse primers labeled with 6-Carboxyfluorescein (6-FAM) on the 5′ end. Concentrations were: 0.5 µM of fluorolabeled reverse primers and 1 µL of each Vβ coamplification product.

### Spectratypes and CDR3 TCR Vβ segments of immunized *Aotus* monkeys

The bp-length and signal intensity of fluorolabeled amplicons were measured in an ABI PRISM 310 Genetic Analyzer using Rox 500 molecular weight markers (both from Applied Biosystems, CA, USA). Spectratypes were analyzed using GeneScan® Analysis Software, assuming that each peak corresponded to a particular TCR CDR3 rearrangement. Spectratype patterns (Gaussian or skewed) were visually evaluated and T-cell oligoclonal expansions were calculated in relative fluorescence intensity units as described elsewhere [46]. Clonally expanded CDR3 segments were amplified by high-fidelity PCR as described above, sequenced and analyzed using Clustal W software.

### Molecular modeling

3D structure models of the *Aotus* αβ TCR–**24112**–HLA-DRβ1*0403 and *Aotus* αβ TCR–**24112**–HLA-DRβ1*0422 molecular complexes was generated by using the crystallographic structure of the human αβ TCR HA1.7 HA peptide–HLA-DR4 (DRA*0101 and DRβ1*0401) as molecular template (PDB code: 1J8H) [20]. Replacements were made on these molecules based on the differences found in the PBR of the high-antibody producer, fully-protected Ao191 monkey on its HLA-DRβ1*0403-like β-chain amino acid sequence (Phe37Tyr, Ser57Asp, Lys60Tyr, Leu61Trp, Ile67Leu, Asp70Gln, Ser74Glu and Gly86Val) as well as in the non-antibody producer, non-protected Ao148 monkey genotyped as HLA-DRβ1*0422-like (Tyr26Phe, Leu67Tyr). Another structure was generated by replacing the Vβ12 family of the HA1.7 TCR CDR3 D region for the ^96^Phe-Leu-Glu-Gly-Gly^100^ sequence found in the D region amino acid sequence of Vβ12 clone 3 derived from the protected Ao191 monkey; Gly105 of the J region was also replaced by Asp. By the same token, the HA 1.7 TCR CDR3 ^96^Arg-Asp-Glu-Glu-Asp^100^ D region was replaced by the ^96^Ser-Thr-Gly-Leu-Pro^100^ sequence found in the Vβ15-clone 5 sequence of the non-antibody producer and non-protected Ao148 monkey.

A conjugate gradient algorithm was applied to minimize energies and build a more energetically favorable model of the complex's position. To obtain the most appropriate model, 6 to 8 simulations with 10,000 iterations were performed for each structure, with each of the Vβ sequences in the complete template (PDB code: 1J8H). Insight II (2000) Biopolymer module software (Accelrys Software Inc., USA), run on an Indigo 2 Station (Silicon Graphics), was used for superimposing the backbones of the original template and the obtained model without further refinements. The rootmean-square deviation between two molecule conformations was then determined.

## Supporting Information

Figure S1 Spectratype analysis of the TCR Vβ repertoire. (A) Representative Gaussian-like profile shown by most TCR Vβ families of Aotus 191 before being immunized with peptide 24112. Only Vβ families 2, 19, 24 and 29 showed a skewed profile in the pre-immune sera. (B) Comparison between TCR Vβ repertoire before (P0 above) and after (PIII below) immunization with peptide 24112. The most notably expanded families were: Vβ12 (in Ao191); Vβ6 (Ao259); Vβ12 (Ao149); Vβ5 (Ao239); Vβ19 (Ao277); Vβ9 (Ao142); Vβ7 (Ao224) and Vβ15 (Ao148), which displayed a Gaussian-like distribution pattern in P0 samples and a skewed pattern in PIII samples from the same monkeys. Due to space limitations, only some examples are shown.
(2.71 MB TIF)

Table S1 Amino Acid Sequences of TCR CDR3β. Some examples of the amino acid sequences of TCR CDR3 sequences expanded in response to immunization with peptide 24112. Sequences were classified according to the immune response induced in Aotus monkeys.
(0.15 MB DOC)

Table S2 Primers designed for amplifying each of the 19 Aotus' TCRβV families. TCRβV family: Names assigned to forward primers amplifying each of the 19 Aotus βV reported to date. Tm: Annealing temperature standardized for each coamplification reaction. βCR: Reverse primer annealing in the TCR β-chain constant region. αCF/αCR: Forward and reverse primers used for TCR α-chain constant region amplification.
(0.05 MB DOC)
