# Supplementary figures and images for: 3D Analysis of the TCR/pMHCII Complex Formation in Monkeys Vaccinated with the First Peptide Inducing Sterilizing Immunity against Human Malaria
Source: PLoS One. 2010 Mar 19;5(3):e9771. doi: 10.1371/journal.pone.0009771 (PMC2841639; doi:10.1371/journal.pone.0009771)

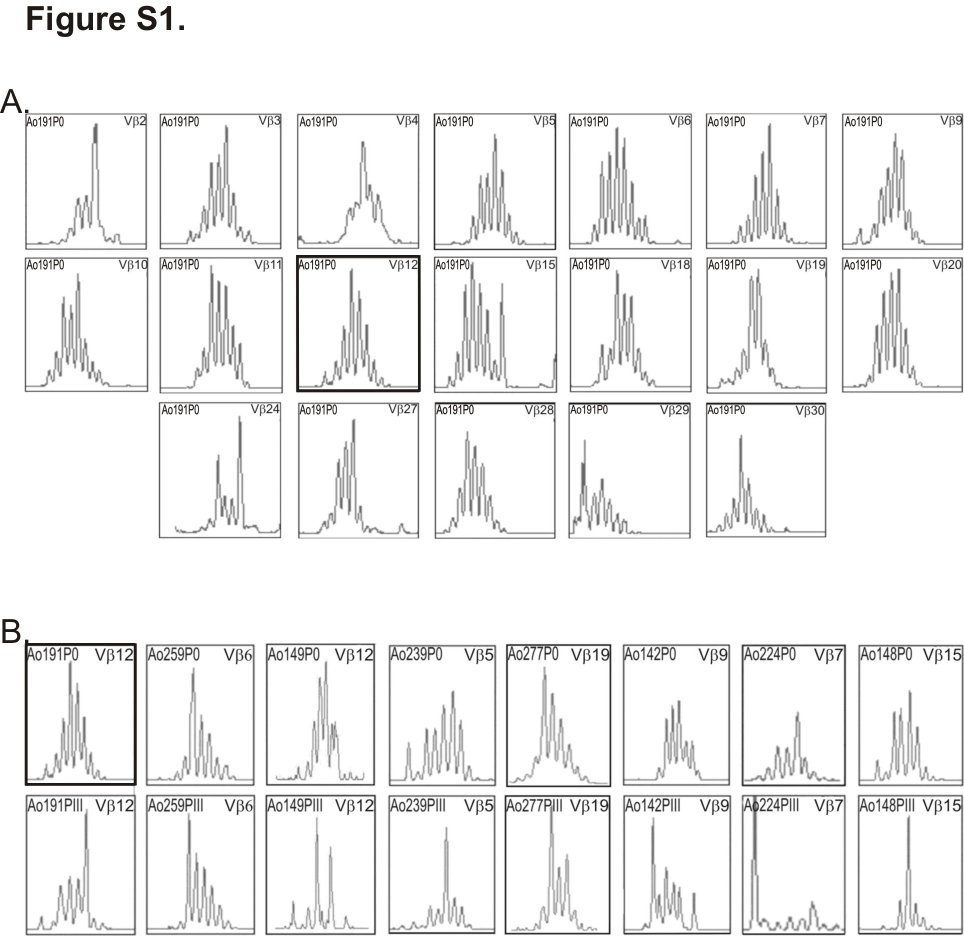

Supplement: Figure S1 — Spectratype analysis of the TCR Vβ repertoire. (A) Representative Gaussian-like profile shown by most TCR Vβ families of Aotus 191 before being immunized with peptide 24112. Only Vβ families 2, 19, 24 and 29 showed a skewed profile in the pre-immune sera. (B) Comparison between TCR Vβ repertoire before (P0 above) and after (PIII below) immunization with peptide 24112. The most notably expanded families were: Vβ12 (in Ao191); Vβ6 (Ao259); Vβ12 (Ao149); Vβ5 (Ao239); Vβ19 (Ao277); Vβ9 (Ao142); Vβ7 (Ao224) and Vβ15 (Ao148), which displayed a Gaussian-like distribution pattern in P0 samples and a skewed pattern in PIII samples from the same monkeys. Due to space limitations, only some examples are shown. (2.71 MB TIF) [file pone.0009771.s001.tif]
