# Supplementary material for: 3D Analysis of the TCR/pMHCII Complex Formation in Monkeys Vaccinated with the First Peptide Inducing Sterilizing Immunity against Human Malaria
Source: PLoS One. 2010 Mar 19;5(3):e9771. doi: 10.1371/journal.pone.0009771 (PMC2841639; doi:10.1371/journal.pone.0009771)
Supplement: Table S1 — Amino Acid Sequences of TCR CDR3β. Some examples of the amino acid sequences of TCR CDR3 sequences expanded in response to immunization with peptide 24112. Sequences were classified according to the immune response induced in Aotus monkeys. (0.15 MB DOC) [file pone.0009771.s002.doc]

**Table S1.**

| **Individual** | **TCRVβ**  **family** | **Clone** | | **Vβ** | **nDn** | **Jβ** | **TCRJβ**  **family** |
| --- | --- | --- | --- | --- | --- | --- | --- |
| **Antibody producing protected individuals** | | | | | | | |
| 191 | 12 | 3 | | CASS | FLEGG | YDYTFGSGTRLT | 1.2 |
|  |  | 4 | | CASS | FDTR | YTFGSGTRLT | 1.2 |
|  |  | 10 | | CASS | LI | TDPLYFGPGTRLT | 2.3 |
|  |  | 9 | | CAS | TFGGG | TEAFFGEGTKLT | 1.1 |
| 259 | 6 | 10 | | CASS | DLLSA | NYDYTFGSGTRLT | 1.2 |
|  |  | 2 | | CAS | RETGS | TDPLYFGPGTRLT | 2.3 |
|  |  | 7 | | CASS | DSLTGSA | EAFFGEGTKLT | 1.1 |
|  |  | 4 | | CAS | **RETE** | NQAQHFGDGTRLS | 1.5 |
|  |  | 5 | | CASS | ACPCG | EQHFGPGTRLT | 2.7 |
| 149 | 12 | 6 | | CASS | LASGS | TDPLYFGPGTRLT | 2.3 |
|  |  | 5 | | CASS | RAV | SYNEQFFGPGTQLT | 2.1 |
|  |  | 11 | | CASS | PDRRR | EKLFFGSGTQLS | 1.4 |
|  |  | 9 | | CASS | PRTEGNTVY | FGEGSRLT | 2.2 |
|  |  | 3 | | CAS | TLQGG | AFFGEGTKLT | 1.1 |
|  |  | 10 | | CAS | TLQGG | AFFGEGTKLT | 1.1 |
| **Antibody producers and non-protected individuals** | | | | | | | |
| 239 | 5 | | 4 | CASS | VGIRD | YDYTFGSGTRLTVV | 1.2 |
|  |  | | 6 | CASS | IRMAG | EAFFGEGTKLTVV | 1.1 |
|  |  | | 7 | CASS | HNRAM | NTEAFFGEGTKLTVV | 1.1 |
|  |  | | 8 | CAS | RPWGG | QFFGPGTRLTVL | 2.1 |
|  |  | | 10 | CASS | LWTGVH | EQHFGPGTRLTVT | 2.7 |
| 277 | 19 | | 1 | CASS | IGL | NTEAFFGEGTKLTVV | 1.1 |
|  |  | | 2 | CASS | TGEG | TEAFFGEDTKLTVV | 1.1 |
|  |  | | 9 | CASS | IFWGSMD | EQFFGPGTRLTVL | 2.1 |
|  |  | | 12 | CASS | TPI | NYDYTFGSGTRLTVV | 1.2 |
|  |  | | 4 | CAS | KEE | NTEAFFGEGTKLTVV | 1.1 |
|  |  | | 8 | CAS | NDGG | NYDYTFGSGTRLTVV | 1.2 |
| **Non-antibody producers or protected individuals** | | | | | | | |
| 148 | 7 | | 1 | CAS | TLGGTGNTVY | FGEGSRLTVV | 2.2 |
|  |  | | 3 | CAS | TGDSNSGNTVY | FGEGSRLTVV | 2.2 |
|  |  | | 4 | CASS | LGRA | NTEAFFGEGTKLTVV | 1.1 |
|  |  | | 6 | CASS | PSGAGS | TDPLYFGPGTRLTVL | 2.3 |
|  |  | | 8 | CASS | LYGPTSGNTVY | FGEGSRLTVV | 2.2 |
|  |  | | 9 | CASS | LDN | YEQHFGPGTRLTVT | 2.7 |
| 148 | 15 | | 4 | CASS | RDRD | FYEQHFGPGTRLTVT | 2.7 |
|  |  | | 5 | CASS | RDEED | YDYTFGSGTRLTVV | 1.2 |
|  |  | | 6 | CASS | RDRD | FYEQHFGPGTRLTVT | 2.7 |
|  |  | | 7 | CASS | RDRD | FYEQHFGPGTRLTVT | 2.7 |
|  |  | | 8 | CASS | RDRD | FYEQHFGPGTRLTVT | 2.7 |
| 148 | 28 | | 2 | CASS | PPGTGRNT | EAFFGEGTKLTVV | 1.1 |
|  |  | | 3 | CA | TKKPGNS | EAFFGEGTKLTVV | 1.1 |
|  |  | | 4 | CASS | SLGNTAH | LFFGEGSRLTVL | 2.2 |
|  |  | | 5 | CASS | IEGVLG | TQYFGAGTRLSVL | 2.4 |
|  |  | | 9 | CASS | LYLRG | NYDYTFGSGTRLTVV | 1.2 |
|  |  | | 10 | CASS | SNGHSG | DYTFGSGTRLTVV | 1.2 |
| 224 | 7 | | 1 | CASS | SDGLY | TDPLYFGPGTRLTVL | 2.3 |
|  |  | | 3 | CAS | RKGTGS | TDPLYFGPGTRLTVL | 2.3 |
|  |  | | 4 | CASS | LMYGA | EAFFGEGTKLTVV | 1.1 |
|  |  | | 5 | CAS | TFSA | NTEAFFGEGTKLTVV | 1.1 |
|  |  | | 6 | CASS | NNN | EQFFGPGTRLTVL | 2.1 |
|  |  | | 8 | CAS | TRTTLGGGI | NTQYFGAGTRLSVL | 2.4 |
| 224 | 10 | | 3 | CASS | ARVW | NTEAFFGEGTKLTVV | 1.1 |
|  |  | | 6 | CASS | DIERT | GHQAQHFGDGTRLSVL | 1.5 |
|  |  | | 7 | CAS | RSGAGSL | ETQYFGPGTRLLVL | 2.5 |
|  |  | | 8 | CASS | DTA | TNEKLFFGSGTQLSVL | 1.4 |
| 224 | 28 | | 1 | CASS | LSILGAG | NEQFFGPGTRLTVL | 2.1 |
|  |  | | 3 | CASS | STTS | SQNTQYFGAGTRLSVL | 2.4 |
|  |  | | 4 | CASS | LSILGAG | NEQFFGPGTRLTVL | 2.1 |
|  |  | | 5 | CASS | STTNSGNTVY | FGEGSRLTVV | 2.2 |
|  |  | | 6 | CASS | LSILGAG | NEQFFGPGTRLTVL | 2.1 |
|  |  | | 8 | CASS | DEPGQGR | EKLFFGSGTQLSVL | 1.4 |
|  |  | | 9 | CASS | FAGDI | EAFFGEGTKLTVV | 1.1 |
|  |  | | 10 | CASS | AGGGL | EQHFGPGTRLTVT | 2.7 |
| 142 | 5 | | 3 | CAS | NTGLADCPC | TDPLYFGPGTRLTVL | 2.3 |
|  |  | | 4 | CASS | HNRAM | NTEAFFGEGTKLTVV | 1.1 |
|  |  | | 7 | CAS | NLVRPE | GGEKLFFGSGTQLSVL | 1.4 |
|  |  | | 8 | CASS | H | WTDPLYFGPGTRLTVL | 2.3 |
|  |  | | 9 | CAS | GQS | VYEQHFGPGTRLTVT | 2.7 |
|  |  | | 10 | CASS | A | GSQAFFGEGTKLTVV | 1.1 |
| 142 | 9 | | 1 | CAS | NLTV | GTDPLYFGPGTRLTVL | 2.3 |
|  |  | | 2 | CAS | NLTV | GTDPLYFGPGTRLTVL | 2.3 |
|  |  | | 4 | CAS | NLMN | QETQYFGPGTRLLVL | 2.5 |
|  |  | | 6 | CAS | NLIGA | EQFFGPGTQLTVL | 2.1 |
|  |  | | 7 | CAS | IGGTS | SYEQHFGPGTRLTVT | 2.7 |
