# Supplementary material for: 3D Analysis of the TCR/pMHCII Complex Formation in Monkeys Vaccinated with the First Peptide Inducing Sterilizing Immunity against Human Malaria
Source: PLoS One. 2010 Mar 19;5(3):e9771. doi: 10.1371/journal.pone.0009771 (PMC2841639; doi:10.1371/journal.pone.0009771)
Supplement: Table S2 — Primers designed for amplifying each of the 19 Aotus' TCRβV families. TCRβV family: Names assigned to forward primers amplifying each of the 19 Aotus βV reported to date. Tm: Annealing temperature standardized for each coamplification reaction. βCR: Reverse primer annealing in the TCR β-chain constant region. αCF/αCR: Forward and reverse primers used for TCR α-chain constant region amplification. (0.05 MB DOC) [file pone.0009771.s003.doc]

| **TCRVβ Family^a^** | **Sequence 5' → 3'** | **Tm^b^** | **GenBank Accession Number** |
| --- | --- | --- | --- |
| Vβ2 | CTCTGAAGATTCAATCCACAAAGC | 55 | AF107760 |
| Vβ3 | CCTGAGTCTCCAGACAAAG | 52 | AY659765 |
| Vβ4 | TCACTTACACCTTCACCTACAC | 55 | AF107746, AF107737 |
| Vβ5 | ACTTCCCTGCTCGATTCTCA | 57 | AF107757, AF107759 |
| Vβ6 | ATTTCCCGCTCAGGCT | 55 | AF107747, AF107752 |
| Vβ7 | GATCCTTCTCCACTCTGA | 52 | AF107736 |
| Vβ9 | ACGATTCTCAGGACAACAGTTC | 55 | AF107756, AF107745 |
| Vβ10 | TTGATGGCTACGTTGTCTC | 52 | AY659766 |
| Vβ11 | CAGAGAGGCTCAAAGGAGTAGA | 55 | AY659767, AY659768 |
| Vβ12 | ATCCAGCCTTCAGAACCCAG | 57 | AF107742 |
| Vβ15 | TGCTTTCTTGACATCCGCTC | 55 | AF107754 |
| Vβ18 | GAGTCAGGAATGCCAAAGGAAC | 57 | AY659770 |
| Vβ19 | TACAGCGTCTCTCGGGAGAA | 57 | AY659771 |
| Vβ20 | CCTGACCTTCTCATCTCTGA | 52 | AY659772 |
| Vβ24 | CCTAAATTCTCCCTGTCCCT | 55 | AF107751 |
| Vβ27 | AATTTCCCCCTGATCCTGG | 55 | AY659774 |
| Vβ28 | AGCACCAACCAGACATCT | 55 | AF107739 |
| Vβ29 | AGCACCAACCAGACATCT | 55 | AY659775, AY659776 |
| Vβ30 | CGATTCTGGCTTCTATCTCTGT | 55 | AY659777 |
| CβR^c^ | TGCTTCTGATGGCTCAAACAC |  |  |
| CαF^d^ | ATATCCAGAACCCTGACCCT |  |  |
| CαR^d^ | CTGGACCACAGCCGCA |  |  |

**Table S2.** **Primers designed for amplifying each of the 19 *Aotus’* TCRβV families. TCRβV family:** Names assigned to forward primers amplifying each of the 19 *Aotus* βV reported to date. **Tm:** Annealing temperature standardized for each coamplification reaction. **βCR:** Reverse primer annealing in the TCR β-chain constant region. **αCF/αCR:** Forward and reverse primers used for TCR α-chain constant region amplification.
